# Caregiver burden, mental health, quality of life and self-efficacy of family caregivers of persons with dementia in Malaysia: baseline results of a psychoeducational intervention study

**DOI:** 10.1186/s12877-024-05221-9

**Published:** 2024-08-05

**Authors:** Hashima E. Nasreen, Marie Tyrrell, Sofia Vikström, Åsa Craftman, Syarifah Amirah Binti Syed Ahmad, Nora Mat Zin, Karimah Hanim Abd Aziz, Noorlaili Binti Mohd Tohit, Mohd Aznan Md Aris, Zarina Nahar Kabir

**Affiliations:** 1 Department of Community Medicine, Faculty of Medicine, International Islamic University Malaysia, Jalan Sultan Ahmad Shah, Kuantan, 25200 Pahang Malaysia; 2 Sophiahemmet Högskola, Valhallavägen 91, 114 86 Stockholm, Sweden; 3 Department of Neurobiology, Care Sciences and Society, Karolinska Institute, Huddinge, Stockholm, SE-141 83 Sweden; 4 Department of Psychiatry, Faculty of Medicine, International Islamic University Malaysia, Jalan Sultan Ahmad Shah, Kuantan, 25200 Pahang Malaysia; 5 Department of Family Medicine, Universiti Kebangsaan Malaysia, Bandar Tun Razak, Cheras, Kuala Lumpur 56000 Malaysia; 6 Department of Family Medicine, Faculty of Medicine, International Islamic University Malaysia, Kuantan, Pahang 25200 Malaysia

**Keywords:** Family caregivers, Persons with dementia, Caregiver burden, Depressive symptoms, Anxiety symptoms, Quality of life, Caregiving self-efficacy

## Abstract

**Background:**

The majority of persons with dementia (PWD) are mainly cared for by their family members in the home. Evidence is however scarce on family caregivers’ psychosocial burden and quality of life in Asian countries including Malaysia. This study describes the baseline data of a telephone-delivered psychoeducational intervention study and examines the determinants of outcome measures (caregiver burden, depressive and anxiety symptoms, quality of life and caregiving self-efficacy) among Malaysian family caregivers to PWD.

**Methods:**

This was a cross-sectional study originated from the baseline survey of a randomized control trial of 121 family caregivers recruited from lists of PWD who were registered at memory and psychiatry clinics in three tertiary care hospitals in Malaysia. The participants were assessed for caregiver burden by the Zarit Burden Interview, depressive and anxiety symptoms by the Hospital Anxiety and Depression Scale, quality of life by the Control, Autonomy, Self-Realization, and Pleasure Scale, and caregiving self-efficacy by the Revised Scale for Caregiving Self-Efficacy.

**Results:**

Prevalence of caregiver burden was 69.4%, depressive symptoms 32.2% and anxiety symptoms 32.2%. Family caregivers to PWD having perceived peer support e.g., social/family/friend/significant other supports were less likely to report caregiver burden, depressive and anxiety symptoms, and more likely to report higher levels of quality of life and caregiving self-efficacy. Being married and PWD’s ability to self-care were associated with lesser likelihood of experiencing caregiver burden, depressive and anxiety symptoms. The other determinants of greater probability of reporting better quality of life were caregivers’ employment and having Islamic faith. Marital status (married), PWD’s ability to self-care, spousal relationship with PWD and shared caregiving process were associated with higher likelihood of reporting caregiving self-efficacy.

**Conclusion:**

Caregiver burden, depressive and anxiety symptoms are prevalent in family caregivers to PWD in Malaysia. Social support and caregiving related factors influence family caregivers’ quality of life and caregiving self-efficacy. Implementing psychoeducational intervention and support in the psychiatry and memory clinics may help improve the psychosocial burden, quality of life and caregiving self-efficacy in family caregivers of PWD.

**Trial registration:**

ISRCTN14565552 (retrospectively registered).

**Supplementary Information:**

The online version contains supplementary material available at 10.1186/s12877-024-05221-9.

## Background

Globally, variations exist in cultural norms and factors such as accessibility, affordability of health and social services which impact reliance of persons with dementia (PWD) on family members to take on a caregiver role [1]. Malaysia as an aging society with a rapid demographic change has, similar to other countries, a growing number of PWD, creating challenges for health and social care [2]. Due to the trajectory of the disease, providing care for a PWD can entail long care hours over a prolonged period impacting on the caregiver and family function and challenging stability of the family [3]. Caring for a family member with dementia can be demanding and stressful. Caregivers to PWD have shown to be at higher risk of developing depression and anxiety than persons caring for family members with other illnesses [4], experience significantly higher levels of caregiver burden [5], low level of quality of life [6] and low level of self-efficacy [7].

Self-reported symptoms of depression and anxiety are common in family caregivers impacting on health and quality of life while caring for a PWD [8]. Women and spousal caregivers are reported as more likely to develop anxiety and depression than their counterparts [4, 8]. Factors that may moderate the presence of anxiety disorder in caregivers to PWD include living with the care recipient, level of dependence of PWD, being a female caregiver, poor relationship with the care recipient and health of the caregiver [9]. According to Wulff et al. [8], the amount of time spent caring for the family member and the severity of person’s dementia impact on the levels of depression and anxiety experienced.

Caregiving responsibilities for a PWD include supporting the person with instrumental activities of daily living such as cooking, shopping, etc., and even basic activities of daily living such as bathing, dressing, mobility, etc. [10, 11]. For a PWD living at home, such support is often provided by family members, 70% of whom are female [12]. According to Tulek et al. [6] high level of caregiver burden is related to low quality of life. Family caregivers’ quality of life can directly impact on the care they provide for the PWD, and how they cope with their own life situation. As dementia is a progressive disease, the family caregiver’s quality of life changes over time in relation to the needs of the caregiver and ability to handle the care recipient’s deteriorating condition.

There are multiple benefits of caring for a PWD which include perception of caring as a meaningful task, a feeling of giving back to the loved one, sense of close family relationship and satisfaction of providing care [5, 13]. However, positive aspects of caregiving can be accompanied by the strains of caregiving leading to a sense of caregiver burden [5]. This in turn can negatively impact caregivers multi-dimensionally, i.e., physically, psychologically, emotionally, behaviourally and financially [14]. Evidence from around the world such as China [15], Indonesia [16], Turkey [6] indicate high levels of caregiver burden for family caregivers to PWD living at home. Several factors are identified as predictors of caregiver burden. These include neuropsychiatric symptoms of PWD [17], functional (cognitive and physical) decline of the PWD cared for which is related to increased number of hours spent in caregiving activities [18], and lack of perceived social support [15, 16]. Spouses to PWD identified that their partners had between five to eight co-existing neuropsychiatric symptoms causing varied levels of distress for the spouses concerned [19]. Family caregivers’ socio-demographic characteristics such as being a woman [16, 20], cohabitation and spousal relationship with the PWD [18] are also identified as significant predictors of caregiver burden.

Several studies suggest that self-efficacy can be a useful concept for explaining variations in caregiving abilities of family caregivers of PWD. Self-efficacy refers to the perception of a person´s capacity to manage responsibilities and tasks successfully and confidently [21]. In relation to family caregiving in dementia, self-efficacy has also been suggested to represent family caregiver’s knowledge and preparedness in managing the challenges of care [22]. Feeling prepared is reported to be associated with low levels of distress [7]. Improvements of caregiver self-efficacy may positively influence caregiver health and well-being [22] and reduce levels of caregiver burden [23]. Likewise, education and skills training, case management, and interventions that target caregiver’s negative emotions are associated with improvements in self-efficacy [24].

Various educational, psychosocial, and multicomponent interventions have demonstrated modest success in improving the quality of life and negative consequences associated with caregiving for persons with dementia [17, 25]. In-person interventions can be difficult for caregivers due to lack of transportation, being homebound, living in a rural setting, time pressures of caregiving, or stigma associated with seeking help [25]. In response to these issues, telephone-based interventions have been reported to improve functioning in caregivers, to persons with dementia, with a variety of clinical problems in USA [25], Australia [26], UK [27], Europe [28] and Hong Kong [29]. Due to its high accessibility and cost-effective implementation, the telephone-delivered support services for dementia caregivers have received global attention. Studies indicated that telephone-based interventions benefited caregivers by means of increasing use of appropriate community services, reducing caregiver burden, depression and distress related to care recipient behaviour, and improving caregiving self-efficacy and quality of life [26, 30].

According to the Alzheimer Disease International [31], the prevalence of dementia in Malaysia was 0.063% in 2005 with the annual incidence rate of 0.020%, and in 2015 was 0.401%. It is projected that this figure will increase to 0.852% and 1.924% in 2030 and 2050, respectively. Like in many Asian countries, family members in Malaysia provide most of the care and support to PWD living in the community [32, 33]. With a view to develop and evaluate a care and support intervention for family caregivers to PWD living in the community in Malaysia, it is imperative to understand their current situation. Hence, this paper describes the baseline data of a telephone-delivered psychoeducational intervention study and explores the associated factors of the outcome measures caregiver burden, depressive and anxiety symptoms, self-efficacy, and quality of life among family caregivers to PWD living at home in Malaysia.

## Methods

### Study design and setting

Data for this cross-sectional study originated from the baseline survey of a randomized control trial (RCT) aimed to assess the efficacy of a telephone-delivered psychoeducational intervention. The intervention was delivered by healthcare professionals (occupational therapists and nurses) to reduce caregiver burden, depressive and anxiety symptoms, and improve caregiving self-efficacy and quality of life among family caregivers to PWD. Participants were recruited from lists of PWD over eight months (August 2022 to March 2023) who were registered at memory and psychiatry clinics of Universiti Kebangsaan Malaysia Medical Centre (UKMMC), Sultan Ahmad Shah Medical Centre (SASMEC) of International Islamic University Malaysia (IIUM) and Hospital Tengku Ampuan Afzan (HTAA). UKMMC (a university tertiary hospital) located in Selangor state, and SASMEC (a university tertiary hospital) and HTAA (a state level tertiary hospital) located in Pahang state in west and east coasts of Malaysia, respectively (see Additional file 1).

### Participants

Participants in this study were family caregivers of PWD who had received a clinical diagnosis of dementia. Participants were included if they were Malaysian citizens, aged 18 years or over, able to read and write Malay, primary caregivers (in case of more than one caregiver), in caregiving role for at least 6 months with at least 4 h caregiving activities per day, and had smartphones. Exclusion criteria included if family caregivers described themselves as having any major acute medical illnesses or if they could not participate throughout the entire study due to lack of time.

**Fig. 1:**
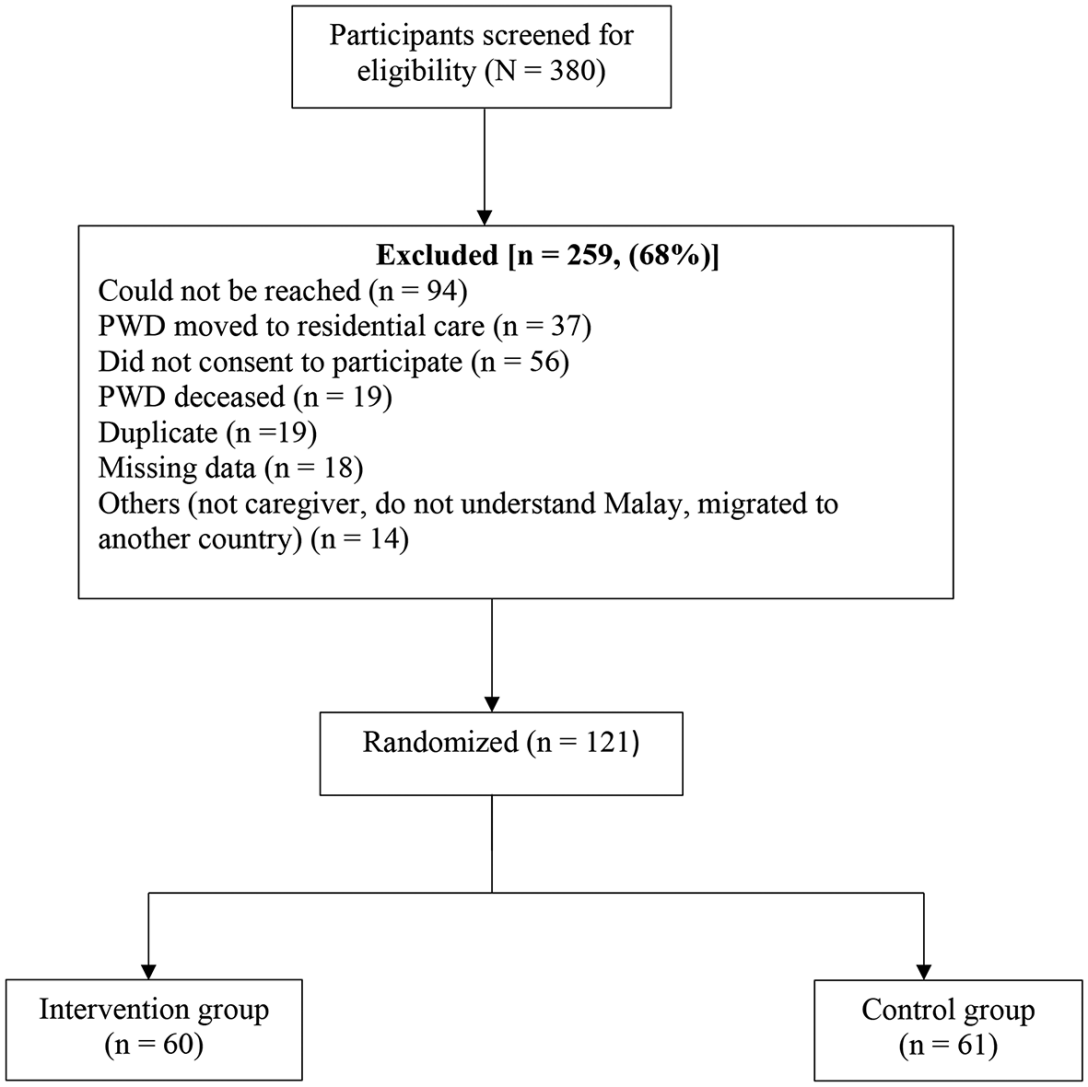
Participants enrollment

Assuming a current improvement rate of 30% in caregiver burden and depressive symptoms, expected net improvement of 25–30% with the intervention [34], a significance level of 5% and power of 80%, the estimated sample size for the RCT was 100 (50 in intervention group and 50 in control group). In case of the secondary outcome i.e., quality of life, considering the mean (SD) in treatment group of 55.58 (17.75) and control of 50.00 (18.37) as a result of intervention [35], a median effect size of 0.5 [36], and a significance level of 5% and power of 80%, resulted in a calculated sample size of 51 (rounded to 50) in each group. In both calculations, the one-sided test was considered. Taking into account a drop-out rate of 20%, the estimated sample size was 60 participants in each group. Initially, 380 family caregivers were screened for eligibility and 121 were assigned to either a psychoeducation group (intervention group) or a control group using a computerized 4-block randomization program (Fig. 1).

### Data collection

Baseline data for the RCT were collected from August 2022 to March 2023. Training of the research assistants included information on the questionnaire and data collection procedures. The trained research assistants were blinded to group assignment, communicated with the family caregivers by telephone to provide detailed information about the purpose and type of study. Further information was given about the psychoeducation intervention including its procedures and protocols, and risks as well as benefits of participating in the study. Only those family caregivers who provided informed consent to participate were enrolled in this study. The research assistants then interviewed the participants at baseline over telephone using structured questionnaire to collect information on their socioeconomic condition (family caregiver’s age, sex, education [primary, secondary or tertiary], occupation [homemaker/unemployed, retired, employed incorporating government and non-government employee], monthly household income [≥RM 10,960 categorized as high-, RM 4,581–RM 10,959 middle- and ≤RM 4.580 low-income]); caregiving information (duration of care, hours of caregiving per day, if the caregiving was shared by other family members, number of family members involved in shared caregiving, caregiver’s relationship with PWD); perceived social support. Perceived social support was assessed by the validated Malay version of the Multidimensional Scale of Perceived Social Support (MSPSS) [37]. MSPSS included 12 items, scored on a 7-point scale from 1 (very strongly disagree) to 7 (very strongly agree). MSPSS comprised of three subscales, including family support (4 items), friends’ support (4 items) and significant others’ support (4 items), a higher score indicating more support [38]. Significant support from others indicated support from a special person who was available when needed, was a real source of comfort. The scale demonstrated good internal consistency in the present study with Cronbach’s alpha of 0.93 on the whole scale and between 0.91 and 0.92 on the three subscales.

### Outcome measures

The outcome measures in the study included caregiver burden, depressive and anxiety symptoms, quality of life and caregiving self-efficacy.

### Caregiver burden

Caregiver burden was measured by the Zarit Burden Interview (ZBI). ZBI is a 22-item inventory, where each item is rated on a 5-point scale (0–4) with the total score ranging from 0 to 88. A higher score indicates greater burden. ZBI assesses caregiver’s subjective feelings of the negative impact of caregiving on emotional and physical health functioning, social life and financial status [39]. The ZBI has been validated in Malaysia, with 70.8% sensitivity and 69.2% specificity using an optimum cut-off score of 22 [40]. This cut-off score was used in this study to categorize family caregivers as having burden. The scale had good internal consistency with Cronbach’s α of 0.92.

### Depressive and anxiety symptoms

Depressive and anxiety symptoms was assessed by the Hospital Anxiety and Depression Scale (HADS) [41]. The HADS is a 14-item scale that requires respondents to endorse a verbal response which is scored as an index of the severity of anxiety or depression. The HADS questionnaire has seven items each for depression and anxiety subscales. Scoring of each item ranges from zero to three, with three denoting highest anxiety or depression level. The Malay version of HADS showed a good sensitivity (90.0% for anxiety and 93.2% for depression) and specificity (86.2% for anxiety and 90.8% for depression) at the cut-off score of 8/9 [41]. Thus, a total subscale score of > 8 points out of a possible 21 denote considerable symptoms of anxiety or depression. The scale presented good reliability in the study with Cronbach’s α of 0.84 for anxiety and 0.78 for depression.

### Quality of life

Quality of life was measured by the validated Malay version of Control, Autonomy, Self-Realization, and Pleasure (CASP-19) scale [42]. The CASP-19 consists of four domains: four items focus on control domain, five items cover the autonomy domain, and five questions each on self-realization and pleasure domains. Each item is rated on a 4-point scale from never (0) to often (3), with a total score ranging from 0 to 57 where higher score indicates better quality of life. CASP-19 showed a good reliability in the study with Cronbach’s α of 0.88.

### Caregiving self-efficacy

The Revised Scale for Caregiving Self-Efficacy (RSCSE) was used to measure caregiving self-efficacy [43]. The RSCSE is a 15-item scale which rates caregivers’ beliefs about their ability to perform caregiving activities according to their recent situation from 0 (cannot do it at all) to 100 (certain they can do it). The scale consists of 3 subscales (5 items per subscale): obtaining respite (item 1 to 5), responding to disruptive patient behaviours (item 6 to 10), and controlling upsetting thoughts (item 11 to 15). The item scores within each subscale are averaged to obtain subscale ratings ranging between 0 and 100. Higher score indicates higher confidence in self-efficacy. The RSCSE has potential uses for both research and clinical purposes. The RSCSE was translated from English to *Bahasa Melayu* and then back to English again by two bilingual public health researchers. The translated Malay version of the instrument was then pretested with the family caregivers of PWD (not included in the study) to check appropriateness of the terminology in *Bahasa Melayu*. RSCSE demonstrated good internal consistency in the present study with the Cronbach’s α for subscales ranged from 0.86 to 0.94.

### Data analysis

Descriptive analyses were performed to report participants’ background characteristics, caregiving information and outcome measures. An independent t-test, $\:\mathcal{X}$^2^ test and Fisher’s exact two-sided p test were conducted to compare means and proportions between groups. As the ZBI, HADS-depression and HADS-anxiety subscales had definitive cut-off points, we used these outcomes as dichotomous and multiple logistic regression were performed to determine their associated factors. We used multiple linear regression (Backward Method) model to assess the determinants of quality of life and caregiving self-efficacy. As the perceived social support and its three subscales were highly multicollinear, separate models were run for each of them. A *p* value of < 0.05 was considered for statistical significance.

## Results

### Sample profile

Table 1 shows the mean age of the 121 family caregivers of PWD included in the study was 52 years, ranging from 23 years to 85 years. 69% of the family caregivers were women and three-quarters were married. Although the mean years of schooling was 13, nearly half of them received tertiary level of education and were employed in private or public sectors. Approximately 56% of the participants were from low-income level and 8% from high-income level with median monthly income of RM 4000 (USD 1 = RM 4.5). About three-fourths of the caregivers were in non-spousal relationship and the majority were PWD’s own children. The family caregivers had been providing care for almost 47.9 months (≅ 4 years) spending about 19 h per day. The majority of PWD (63%) were women with the mean age of 75 years and 56% were able to take care of themselves (self-care). 60% of the caregivers stated that they received support from other family members in providing care for PWDs. The average scores were 59.3 for perceived social support, 21.1 for family support, 16.1 for friend support and 21.7 for significant other support.

**Table 1:** Sample characteristics of family caregivers of persons with dementia

	Total sample *N* = 121
**Family caregivers**	
Age (years), Mean (SD)	51.6 (12.7)
Sex (%) Male Female	30.6 69.4
Religion (%) Muslim Hindu/Buddhist/Christian	66.9 33.1
Education (%) Primary Secondary Tertiary	13.2 39.7 47.1
Years of studies, Mean (SD)	12.9 (3.4)
Marital status (%) Unmarried Married Divorced/widowed	18.2 73.6 8.2
Occupation (%) Employed Homemaker/unemployed Retired	54.5 35.5 10.0
Monthly HH income (RM), Median (IQR)	4,000 (69,500)
**Caregiving information**	
Length of caregiving (months), Mean (SD)	47.9 (42.8)
Hours of caregiving/day, Mean (SD)	18.6 (6.9)
Shared caregiving by other family members (%)	60.3
Number of persons involved in shared caregiving, Mean (SD)	1.3 (1.5)
Relationship with person with dementia (%) Spouse Adult child In-laws	27.3 62.8 9.9
**Persons with dementia**	
Age (year), Mean (SD)	75.2 (10.1)
Sex (%) Male Female	37.2 62.8
Able to self-care (%)	56.2
**Social support**,** Mean (SD)**	
Social support	59.3 (17.1)
Family support	21.2 (6.4)
Friend support	16.1 (7.3)
Significant other support	21.7 (6.5)

### Descriptives of the outcome measures

Of the 121 caregivers, 84 had ZBI score ≥22, indicating that the point prevalence of caregiver burden was 69.4% (CI_95%_ 59.5 − 79.3%) with a mean score of 41.8 (SD 14.5). Similarly, the point prevalences of depressive and anxiety symptoms were 32.2% (CI_95%_ 17.5 − 46.9%), mean score10.9 (SD 2.3) and 36.4% (CI_95%_ 34.3 − 38.5%), mean score 11.3 (SD 3.3), respectively. The mean score was 40.3 (10.5) for quality of life and 71.8 (28.9), 71.1 (23.8) and 76.8 (19.3) on the three subscales of caregiving self-efficacy (Table 2).

**Table 2:** Percentages and mean scores of outcome variables

	Total sample *N* = 121	Min–max scores
**Caregiver burden (%)**	69.4	
**Depressive symptoms (%)**	32.2	
**Anxiety symptoms (%)**	36.4	
**Caregiver burden**,** Mean (SD)**	32.9 (18.4)	1–77
**HADS-depression**,** Mean (SD)**	5.5 (4.2)	0–15
**HADS-anxiety**,** Mean (SD)**	6.2 (4.7)	0–21
**Quality of life**,** Mean (SD)**	40.3 (10.5)	15–57
**Self-efficacy**,** Mean (SD)**		
Self-care and obtaining respite, Mean (SD)	71.8 (28.9)	0-100
Responding to disruptive patient behavior, Mean (SD)	71.1 (23.8)	0-100
Controlling upsetting thoughts about caregiving, Mean (SD)	76.8 (19.3)	10–100

### Determinants of the outcome measures

Table 3 reveals the adjusted odds ratio (OR) obtained in the multiple logistic regression models showed that family caregivers were less likely to report caregiver burden, depressive and anxiety symptoms if they were married and if the PWDs were able to self-care. Those with higher perceived social support, particularly family support were also less likely to report caregiver burden, depressive and anxiety symptoms. Support from a significant other was found to be significantly associated with caregiver burden and depressive symptoms but not with anxiety symptoms. Moreover, family caregivers in older age and with longer caregiving duration were less likely to report depressive symptoms. Hosmer and Lemeshow tests for caregiver burden (Chi-square = 4.386, *p* = 0.821), depressive symptoms (Chi-square = 4.885, *p* = 0.770) and anxiety symptoms (Chi-square = 4 = 10.157, *p* = 0.254) indicated that models fitted the data well.

**Table 3:** Multiple logistic regressions showing factors affecting caregiver burden, depressive and anxiety symptoms in family caregivers of PWD

	Caregiver burden				Depressive symptoms				Anxiety Symptoms			
	Burdened	Not Burdened	OR	95% CI	Depressed	Not Depressed	OR	95% CI	Anxious	Not Anxious	OR	95% CI
Caregiver’s age (year)^+^	51.3 (2.6)	52.3 (13.0)	−	−	50.4 (13.0)	52.2 (12.5)	0.93*	0.87–0.99	49.5 (12.9)	52.7 (12.5)	−	−
Marital status												
Unmarried/ divorced/ widowed	28 (33.3)	4 (10.8)	1		15 (37.5)	17 (21.0)	1		17 (40.5)	15 (19.0)	1	
Married	56 (66.7)	33 (89.2)	0.23*	0.06–0.82	25 (62.5)	64 (79.0)	0.24*	0.07–0.83	25 (59.5)	64 (81.0)	0.24*	0.08–0.71
PWD able to self-care												
No	43 (51.2)	10 (27.0)	1		22 (55.0)	31 (38.3)	1		23 (54.8)	30 (38.0)	1	
Yes	41 (48.8)	27 (73.0)	0.29*	0.11–0.76	18 (45.0)	50 (61.7)	0.34*	0.13–0.92	49 (62.0)	19 (45.2)	0.33*	0.13–0.84
Duration of care (month)^+^	49.5 (39.7)	44.3 (49.6)	−	−	40.0 (28.1)	51.8 (48.1)	0.98*	0.97–0.99	45.0 (38.3)	49.5 (45.2)	−	−
Shared caregiving												
No	36 (42.9)	12 (32.4)			18 (45.0)	30 (37.0)			21 (50.0)	27 (34.2)	1	1
Yes	48 (57.1)	25 (67.6)	−	−	22 (55.0)	51 (63.0)	−	−	21 (50.0)	52 (65.8)	0.28*	0.09–0.89
Social support^+^	56.9 (17.8)	64.6 (14.5)	0.96*	0.93–0.99	51.4 (17.6)	63.1 (15.6)	0.94**	0.91–0.98	52.6 (19.2)	62.8 (14.8)	0.96*	0.93–0.99
Family support^+^	20.3 (6.6)	23.8 (5.2)	0.89*	0.81–0.98	19.3 (7.0)	22.4 (5.8)	0.91*	0.83–0.99	19.0 (7.0)	22.7 (5.6)	0.91*	0.85–0.99
Friend support^+^	15.9 (7.5)	16.7 (7.0)	−	−	13.1 (7.3)	17.6 (6.9)	0.90**	0.84–0.97	14.3 (8.0)	17.1 (6.8)	−	−
Significant other support^+^	20.7 (6.7)	24.1 (5.2)	0.90*	0.82–0.99	19.1 (7.1)	23.1 (5.8)	0.89*	0.81–0.98	19.2 (7.1)	23.1 (5.8)	−	−

Models were adjusted for caregiver’s sex, religion, years of study & occupation; PWD’s age & sex; hours of care per day; and number of family caregiver

^+^Continuous variable

*p < 0.05

**p < 0.01

Table 4 presents the adjusted linear regression models on the continuous outcome variables showed that the higher the perceived social support including family support, friend support and significant other support, the higher the quality of life and caregiving self-efficacy for obtaining respite among the family caregivers. However, friend support was not identified as a determinant for self-efficacy for responding to disruptive patient behaviors and self-efficacy for controlling upsetting thoughts about caregiving. In addition, family caregivers who were employed and identified themselves as Muslim were more likely to report better quality of life. Caregiving self-efficacy for responding to disruptive patient behavior was more likely to be reported if the family caregivers were married, and self-efficacy for controlling upsetting thoughts about caregiving if PWDs were able to care for themselves (self-care). Family caregivers who were spouses and shared caregiving were more likely to report caregiving self-efficacy for obtaining respite and controlling upsetting thoughts about caregiving. Moreover, duration of care was negatively associated with self-efficacy for obtaining respite.

**Table 4:** Multiple linear regressions showing factors associated with quality of life and caregiving self-efficacy among family caregivers of PWD

	Quality of life		Caregiving self-efficacy					
			Obtaining respite		Responding to disruptive behaviours		Controlling upsetting thoughts	
	B (SE)	*p* value	B (SE)	*p* value	B (SE)	*p* value	B (SE)	*p* value
Married caregiver (Married = 1, unmarried/ divorced/widowed = 0)	−	−	−	−	9.68(4.62)	0.038	−	−
Muslim caregiver (Muslim = 1, non-Muslim = 0)	4.05(1.82)	0.028	−	−	−	−	−	−
Working caregiver (employed = 1, unemployed/ retired = 0)	3.92(1.70)	0.023	−	−	−	−	−	−
Relationship with PWD (spouse = 1, child/in-law = 0)	−	−	13.55(5.83)	0.022	−	−	8.21(3.94)	0.040
Shared caregiving (yes = 1, no = 0)	−	−	14.10(4.85)	0.004	−	−	7.55(3.58)	0.037
Duration of care	−	−	-0.16(0.05)	0.003	−	−	−	−
Age of PWD	−	−	0.70(0.24)	0.005	−	−	−	−
PWD able to self-care (yes = 1, no = 0)	−	−	−	−	−	−	6.67(3.28)	0.045
Social support score	0.26(0.05)	< 0.001	0.71(0.14)	< 0.001	0.36(0.12)	0.003	0.38(0.10)	< 0.001
	*R* = 0.55, R^2^ = 0.31		*R* = 0.60, R^2^ = 0.36		*R* = 0.38, R^2^ = 0.14		*R* = 0.44, R^2^ = 0.20	
Family support	0.64(0.13)	< 0.001	1.92(0.37)	< 0.001	1.12(0.32)	0.001	1.10(0.25)	< 0.001
	*R* = 0.53, R^2^ = 0.28		*R* = 0.60, R^2^ = 0.37		*R* = 0.36, R^2^ = 0.11		*R* = 0.44, R^2^ = 0.20	
Friend support	0.38(0.12)	0.002	1.15(0.33)	0.001	−	−	−	−
	*R* = 0.51, R^2^ = 0.26		*R* = 0.53, R^2^ = 0.28		−		−	
Significant other support	0.66(0.13)	< 0.001	1.72(0.36)	< 0.001	0.98(0.32)	0.003	1.13(0.26)	< 0.001
	*R* = 0.55, R^2^ = 0.30		*R* = 0.58, R^2^ = 0.33		*R* = 0.38, R^2^ = 0.14		*R* = 0.47, R^2^ = 0.22	

Models were adjusted for caregiver’s age & years of study; PWD’s age & sex; duration of care; hours of care per day; and number of family

NB. Because of high multi-collinearity between the three subscales of multidimensional scale of perceived social support, independent model was run, and different R and R^2^ were reported for each of them

## Discussion

The current study examined baseline measures of a telephone-delivered psychoeducational intervention for family caregivers to PWD in Malaysia. The majority of the family caregivers in this study were married [20] and women, a similar pattern to that reported in Asia [44] and other parts of the world [45, 46]. Although spouses are often reported to take on the primary role as caregivers to PWD in countries, such as Shanghai, China [15] and Australia [20], most of the caregivers in this study were adult children. The majority of participants shared caregiving responsibilities with other family members over an average of four years. This timeframe concurs with similar studies from e.g. Argentina [45], United States of America [47], Canada [48], and Iran [5]. Where caregiving became a shared responsibility, women in mixed-gender sibling group, are shown to take the lead in caregiving [48].

In this study, the mean caregiving hours were on average 18 h per day. This figure exceeds data from European countries with a mean of 6 h/day [49], however, in line with a study from Malyasia 15 h caregiving was reported per day [50]. Family caregivers reported in the current study how their engagement in caregiving extended beyond caregiving activities. This perspective is potentially influenced by their cohabitation with the PWD and an ongoing sense of responsibility throughout the day. This aligns with findings from an Austrian study, showing that family caregivers feel the need to be constantly available and on guard due to the dependency of the PWD [51].

In the current study, the prevalence of caregiver burden was almost 70% compared to a meta-analysis where the prevalence ranged from 35.8 to 88.5% with a pooled prevalence of 62.5% [52]. The mean caregiver burden score of 32.9 in this study mirrors that of international studies using the ZBS, with scores of: 30.7 in Australia [20], 34.1–37.4 in Hong Kong, China [29] and lower scores in India of 47.9 [44]. In this study, family caregivers who were married were less likely to experience burden possibly due to support from their spouses in caregiving responsibilities. Family caregivers with higher perceived social support, particularly from family and significant others, were less likely to experience caregiver burden. A study from Nepal [53] suggested that the majority of family caregivers (88%) in their study who experienced little to no caregiver burden was attributed to cultural factors such as filial piety and responsibilities (Khanal & Chalise, 2020). The results of this study indicated the higher the level of self-care among PWD the less likely family caregivers reported burden of care. This has been attributed to how increasing function dependency due to severity of dementia entails longer caregiving hours, thereby contributing to greater caregiver burden [17].

In this study the prevalence of caregiver depressive and anxiety symptoms were 32% and 36%, respectively. Similar findings were reported by Collins and Kishita [52] with informal caregivers had a pooled prevalence of depression of 31% and a 32% pooled prevalence of anxiety [54]. Family caregivers were less likely to report depressive and anxiety symptoms if, they were married, if the PWD was able to care for self, and if social support was available, especially from family and significant others. Finally, older family caregivers who provided care over a long period of time reported less symptoms. An important determinant in protecting family caregivers from depression and anxiety, in an Asian population, is the caregiver´s perceived sense of control over the situation (also known as mastery) and level of competence. These factors were independently associated with negative outcomes associated with caregiver depression and anxiety [55].

The availability and accessibility of support in societies with mostly family caregivers are positively related to quality of life [1]. Consistent with other research [2, 56], the results indicate that family caregivers reporting a higher social support network from e.g. family, friends and significant others were more likely to report better quality of life. In addition, improved quality of life was more likely to be reported by family caregivers who were employed and identified themselves as Muslim. This can be explained by a perception of the importance of culture and belief as a family caregiver. As suggested in a review from China [57], family caregivers showed lower depressive symptoms most likely due to the perception of fulfilling society’s cultural requirements to take care of family members.

Similar to findings by Tan et al. [58], this study showed that older family caregivers were likely to report a high sense of self-efficacy in terms of controlling upsetting thoughts, partly due to their life experiences and age. This might partly explain why spouses of PWD were more likely to report high self-efficacy in terms of obtaining respite and controlling negative emotions, in comparison to their children, both in this study and Colloby et al. [59]. Familiarity of routines and aspects of relationship may act as a buffer for some initial challenges experienced by older family caregivers [59]. Both Tew et al. [60] and Tan et al. [58] mirror this study´s results, that caregivers living with the care recipient demonstrate lower levels of self-efficacy than others. This study shows that shared caregiving was positively associated with obtaining respite and controlling upsetting thoughts. According to Steffen et al. [43] increased social support, enhances the likelihood that the family caregiver will find someone to care for the PWD if respite is needed. Participants in this study scored notably higher mean scores (all above 71) of self-efficacy in all three domains of the Revised Scale For Caregiving Self-Efficacy; Obtaining Respite, Responding to Disruptive Patient Behaviors, and Controlling Upsetting Thoughts, compared to Steffen et al. [43]. Steffen, et al. [43] found that family caregivers with higher perceived social support reported greater care-giver efficacy. This aligns with the Social Cognitive Theory which suggests a bidirectional relationship between perceived coping efficacy and social support [21].

The current study has used locally validated instruments to measure caregiver burden, depressive and anxiety symptoms, and quality of life except for the caregiving self-efficacy. Translation and back translation were done on the RSCSE scale by two bi-lingual researchers, to help minimize error and inconsistencies. As the instrument has not been used in Malaysia before, the results should be interpreted with caution. The ZBI is known to have a high rate of false positive as the detection of levels of experienced burden can be impeded by respondent’s gender and culture. However, higher sensitivity of 70.8% with a lower but reasonable specificity of 69.2% at the recommended cut-off score in this study, indicated that a false negative rate is less desired than a false positive case [40]. Duration of caregiving per day were reported subjectively according to respondents’ perceptions may introduce bias through over-estimation. Although telephone interviews have been identified as cost-effective and time-efficient, the greatest challenge with this type of interview is the loss of several communication cues, distractions, and technical difficulties. However, by allowing flexibility and breaking the interview into multiple sessions can help overcome such challenges.

## Conclusion

This study contributes to current research on protective factors that enhance sustainable support for family members caring for a PWD in Malaysia. This indicates a necessity of implementing a psychoeducational intervention for the family caregivers to a PWD in the memory, geriatric, and psychiatry clinics in Malaysia. Given the time and efforts associated with caring for PWDs, a telephone delivered intervention has the potential to be highly accessible for the family caregivers. Such an intervention needs to be culturally adopted and its efficacy examined in reducing psychosocial burden and improving quality of life and caregiving self-efficacy in family caregivers of PWDs in Malaysia. Further research is required in Asian countries to gain an understanding of cultural and economic factors and gender roles when designing support programmes for family caregivers in a community setting.

### Electronic supplementary material

Below is the link to the electronic supplementary material.

Supplementary Material 1

## Data Availability

The dataset generated and analysed during the current study are not publicly available due to confidentiality issues but are available from the corresponding author on reasonable request.

## Declarations

### Authors’ information

Hashima E Nasreen, MBBS, MPH, PhD, Associate Professor of Epidemiology, Department of Community Medicine, Faculty of Medicine, International Islamic University Malaysia, Kuantan Campus, Pahang, Malaysia.

Marie Tyrrell, Associate Professor, Sophiahemmet Högskola, Valhallavägen, Stockholm, Sweden.

Sofia Vikström, PhD, Senior Lecturer, Department of Neurobiology, Care Sciences and Society, Division of Occupational Therapy, Karolinska Institutet, Stockholm, Sweden.

Åsa Craftman, PhD, Senior Lecturer, Department of Neurobiology, Care Sciences and Society, Division of Nursing, Karolinska Institutet, Stockholm, Sweden.

Syarifah Amirah Binti Syed Ahmad, Candidate for Masters in Medical Science, Department of Community Medicine, Faculty of Medicine, International Islamic University Malaysia, Kuantan Campus, Pahang, Malaysia.

Karimah Hanim Abd Aziz, MD, DrPH, Assistant Professor, Department of Community Medicine, Faculty of Medicine, International Islamic University Malaysia, Kuantan Campus, Pahang, Malaysia.

Noorlaili Binti Mohd Tohit, MD, MMeD, PhD, Associate Professor, Department of Family Medicine, Universiti Kebangsaan Malaysia, 56000 Bandar Tun Razak, Kuala Lumpur, Malaysia.

Nora Mat Zin, MD, MMeD, Associate Professor, Department of Psychoiatry, Faculty of Medicine, International Islamic University Malaysia, Kuantan Campus, Pahang, Malaysia.

Mohd Aznan Md Arid, MBBS, MMeD, Professor, Department of Family Medicine, Faculty of Medicine, International Islamic University Malaysia, Kuantan Campus, Pahang, Malaysia.

Zarina Nahar Kabir, PhD, Associate Professor of Public Health and Principal Researcher, Department of Neurobiology, Care Sciences and Society, Division of Nursing, Karolinska Institutet, Stockholm, Sweden.

## Abbreviations

PWD: Persons with dementia
RCT: Randomized control trial
MSPSS: Multidimensional Scale of Perceived Social Support
ZBI: Zarit Burden Interview
HADS: Hospital Anxiety and Depression Scale
CASP: Control Autonomy Self-Realization and Pleasure Scale
RSCSE: Revised Scale for Caregiving Self-Efficacy
SD: Standard deviation
