# Supplementary material for: Caregiver burden, mental health, quality of life and self-efficacy of family caregivers of persons with dementia in Malaysia: baseline results of a psychoeducational intervention study
Source: BMC Geriatr. 2024 Aug 5;24:656. doi: 10.1186/s12877-024-05221-9 (PMC11301828; doi:10.1186/s12877-024-05221-9)
Supplement: Supplementary file 1 — Supplementary Material 1 [file 12877_2024_5221_MOESM1_ESM.docx]

**Additional file 1**

**Number of participants by recruitment methods**

| Recruitment method | Screened  N = 380 (%) | Enrolled  N = 121 (%) |
| --- | --- | --- |
| Hospital list (existing cases) | 260 (68%) | 81 (67%) |
| Family caregivers at the memory clinic | 32 (8%) | 15 (12%) |
| Hospital appointment list (new cases) | 86 (23%) | 23 (19%) |
| Professional/community referral | 2 (0.01) | 2 (0.02%) |
